# Deforestation and stream warming affect body size of Amazonian fishes

**DOI:** 10.1371/journal.pone.0196560

**Published:** 2018-05-02

**Authors:** Paulo Ilha, Luis Schiesari, Fernando I. Yanagawa, KathiJo Jankowski, Carlos A. Navas

**Affiliations:** 1 Departamento de Ecologia, Instituto de Biociências, Universidade de São Paulo, São Paulo, São Paulo, Brazil; 2 Gestão Ambiental, Escola de Artes, Ciências e Humanidades, Universidade de São Paulo, São Paulo, São Paulo, Brazil; 3 US Geological Survey, Upper Midwest Environmental Sciences Center, La Crosse, Wisconsin, United States of America; 4 Departamento de Fisiologia, Instituto de Biociências, Universidade de São Paulo, São Paulo, São Paulo, Brazil; University of Tasmania, AUSTRALIA

## Abstract

Declining body size has been suggested to be a universal response of organisms to rising temperatures, manifesting at all levels of organization and in a broad range of taxa. However, no study to date evaluated whether deforestation-driven warming could trigger a similar response. We studied changes in fish body size, from individuals to assemblages, in streams in Southeastern Amazonia. We first conducted sampling surveys to validate the assumption that deforestation promoted stream warming, and to test the hypothesis that warmer deforested streams had reduced fish body sizes relative to cooler forest streams. As predicted, deforested streams were up to 6 °C warmer and had fish 36% smaller than forest streams on average. This body size reduction could be largely explained by the responses of the four most common species, which were 43–55% smaller in deforested streams. We then conducted a laboratory experiment to test the hypothesis that stream warming as measured in the field was sufficient to cause a growth reduction in the dominant fish species in the region. Fish reared at forest stream temperatures gained mass, whereas those reared at deforested stream temperatures lost mass. Our results suggest that deforestation-driven stream warming is likely to be a relevant factor promoting observed body size reductions, although other changes in stream conditions, like reductions in organic matter inputs, can also be important. A broad scale reduction in fish body size due to warming may be occurring in streams throughout the Amazonian Arc of Deforestation, with potential implications for the conservation of Amazonian fish biodiversity and food supply for people around the Basin.

## Introduction

The impacts of deforestation on tropical stream fishes have been relatively well-documented, especially those mediated by siltation, and changes in physical habitat and food resources [1; 2; 3; 4; 5; 6; 7; 8]. However, little information exists concerning fish responses to changes in stream thermal regimes driven by forest clearing. Forest cover has a major impact on local temperatures by intercepting and reflecting incident solar radiation and through the process of evapotranspiration, which dissipates heat while promoting water flow from deep soil layers to the atmosphere [9; 10]. In the “Amazonian Arc of Deforestation”, for example, complete forest clearing increases mean air temperatures by five Celsius degrees [11], and Macedo et al. [12] demonstrated that mean daily temperatures in streams in deforested watersheds were three to four degrees higher than streams in forested watersheds. This magnitude of temperature change is greater than the worst-case scenarios for global climate warming anticipated by the year 2100 [13].

Temperature is one of the most important environmental factors affecting the performance and distribution of organisms, their interactions, and the functioning of ecological communities [14; 15]. For this reason, perturbation in natural thermal regimes may cause pervasive impacts on biological systems. Changes in phenology and geographic distribution of species are recognized as the two most general biological responses to increasing temperatures associated with climate change [16; 17]. More recently, however, body size reductions have been suggested as a third general response of organisms to increasing temperatures, from evidence amassed in taxa as diverse as bacteria, algae, higher plants, crustaceans, fish, amphibians, birds and mammals [16; 17; 18; 19]. Because body size affects virtually every aspect of an organism´s biology–from its physiology (metabolic costs, ability to endure shortages of food, water and oxygen) to life history (longevity, fecundity, mating success) and biotic interactions (ability to avoid natural enemies and deal with competitors) [20; 21]–temperature-driven body size reductions could raise significant concerns for the conservation of biological biodiversity.

A general, negative effect of increased temperatures on body sizes could manifest across levels of biological organization from communities to individuals [18]. That is, a community-level reduction in mean body sizes (the “community body size shift hypothesis”) could result from either or both an increase in the number or relative abundance of small-sized species (a community-level response termed the “species shift hypothesis”—historically known as Bergman’s rule) and a reduction in the mean body size of its constituent populations (a population-level response termed the “population body size shift hypothesis”—historically known as James’ rule). If present, such population-level body size reductions could again result from either or both an increase in the proportion of juveniles (a population-level response termed the “population age-structure shift hypothesis”) and a decrease in size-at-age (an individual-level response termed “the size-at-age shift hypothesis”, more broadly known as the “temperature-size rule”). Various mechanisms may mediate this temperature-size response, including a high thermal sensitivity of catabolic rates relative to anabolic rates, a high thermal sensitivity of differentiation (and therefore development) relative to growth rates, or a high thermal sensitivity of environmental factors that can limit individual growth [22; 23]. Although the population age-structure shifts hypothesis does not correspond to any thermal rule, it is suggested as an alternative explanation if a body size shift in a population is observed without a corresponding shift in size-at-age [18; 24].

Among natural biological systems, aquatic ecosystems in tropical regions possess a set of characteristics that increase their potential to be impacted by warming-induced reductions in body size. One possibility requiring further validation is that temperature-size responses are greater in aquatic than terrestrial species due to the lower oxygen availability and greater effort required to increase oxygen uptake in water [23]. The hypothesis that oxygen supply is limiting to growth and underlies global warming driven size reductions, however, has been disputed by several authors (e.g., [25; 26; 27]). Besides that, maintenance costs may escalate faster in warmer climates due to the exponential increase in metabolic rate with temperature [28]. Finally, low elevation tropical species typically have an upper individual thermal limit for survival closer to the optimum temperature than their temperate counterparts [29; 30] because they evolved in an environment experiencing low seasonal variation in temperature (stenothermal species). In contrast, high-elevation tropical species [31] or temperate species are generally adapted to a wider range of temperatures (eurythermal species) given the large seasonal variation in temperature in the environments where they evolved [16].

Considering that 7.8 million hectares of native habitats are deforested every year (average for 1990–2015), most of which is in mega-diverse agricultural frontiers around the wet tropics [32], warming-induced reductions in body size could have enormous ecological implications. However, to the best of our knowledge, no studies have evaluated whether environmental warming driven by deforestation can induce reductions in body size. Our aim in this study was to evaluate changes in fish body size from individuals to assemblages under warming driven by deforestation. We focused on headwater streams of the Xingu River in the Southeastern portion of the Amazonian Arc of Deforestation, the same region where large-scale stream temperature shifts were observed by Macedo et al. [12]. After confirming that deforestation led to stream warming, we tested the community body-size shift hypothesis and the population body-size shift hypothesis by means of field surveys, and a specifically designed laboratory experiment to isolate the effects of temperature on body size in the most common fish species in the region. We predicted that mean fish body mass would be reduced in deforested streams relative to forested streams at the assemblage and population levels, and that high temperatures similar to those occurring in deforested streams would negatively affect fish growth.

## Materials and methods

### Study area

We conducted this study in the region of the headwaters of the Xingu River in southeastern Amazonia, one of the largest southern tributaries of the Amazon River. Regional climate is “Aw” according to Köppen´s classification, i.e., savanna tropical climate with distinct dry (May-September) and wet (October-April) seasons. Average annual precipitation is 1900 mm, two-thirds of which is concentrated between December and March (Grupo AMaggi, pers. comm.). Our surveys were conducted at Tanguro Ranch (between longitudes 52°23'30"W and 52°18'50"W, and latitudes 13°9'12"S and 12°41'40"S), in the municipality of Querência, in the eastern part of the state of Mato Grosso, Brazil. At the time of sampling (2013), 60% percent of the 83,000 ha of Tanguro Ranch were covered with evergreen seasonal transitional forest between the ombrophilous rainforests in the north and the woody cerrados in the south of the Xingu Basin [33]. The remaining 40 percent were covered with intensively cultivated soybean fields.

We sampled three first-order streams draining forested watersheds (“forested” streams—“APP2”, and “APP2A”, tributaries to the Darro River watershed; and “APPM”, a tributary to the Tanguro River) and three streams draining deforested watersheds (“deforested” streams—“TAN1”, “TAN2”, and “TAN3”, tributaries to the Darro River watershed). None of the first-order streams we sampled were tributaries of the same second-order stream (see S1 Fig for a map of the location of study area and fish sampling sites). All streams in deforested watersheds had a similar land use history (they were converted from forest to pasture in the early 1980s, and from pasture to soybean between 2003 and 2008) and riparian buffers 70-150m wide covered by grasses and/or early secondary forests (average canopy cover 21%) [31]. Sampling sites at forest streams had average canopy cover of 86% [34] and watersheds almost entirely covered by native forests.

### Stream water temperature and general habitat characterization

The assumption that deforestation led to stream warming was validated by field samplings of water temperatures. On three different dates in the late rainy season (between 24-Jan-2013 and 1-Feb-2013), we measured water temperatures at hourly intervals (from 9 to 19 hours) in 10 sites regularly distributed within the first 2 km of each stream (i.e., every 220 m from the headwater). The order of stream and site sampling was varied haphazardly in each sampling date so as not to bias the measurements. All measurements were taken with a hand-held temperature electrode (ECTestr—Eutech Instruments) midway through the water column in the middle of the channel, except in impounded reaches (there were no impoundments in forest streams, but all deforested streams had one or more in their course), where temperature was measured one meter from the margin.

In addition, we characterized stream habitat of the 50m-long reaches where fish sampling was performed in October and November 2013 (early rainy season). Replicated measurements of 10 environmental variables were taken at 6 perpendicular transects crossing stream reaches equidistantly (each 10 m). Measured variables were: channel width and depth, proportional substratum cover (categorized as coarse benthic organic matter, fine benthic organic matter, sand, macrophytes, and grasses), canopy cover, and water conductivity and pH. Channel width was measured as the transversal distance between stream margins, and channel depth was measured at mid-channel, and between mid-channel and each margin. Proportional substratum cover was measured at mid-channel, at each margin, and between mid-channel and each margin. Canopy cover was estimated by analysis of photos taken close to water level in mid-channel towards canopy (classified in the categories of 0–10%; 10–25%; 25–50%; 50–75%; 75–100%). Conductivity and pH were measured with electrodes (Eutech Instruments) midway through the water column in the middle of the channel.

### Stream fish sampling

To test the community body size shift hypothesis and the population body size shift hypothesis, we sampled fish assemblages in three 50m-long stream sections regularly spaced over the first two kilometers of each stream (0, 1 and 2 km from the headwater). After extensive field trials in the study area, dipnetting was found to be the only sampling methodology appropriate for fish sampling in the narrow, shallow streams typically rich in coarse woody debris, where the low water conductivity (~10 uS/cm) prevented effective electrofishing, and gill and seine netting were impossible. Fish sampling was done by two people, who thoroughly sampled each stream reach with dipnets (1mm mesh size) for a period of 50 minutes in October and November 2013. All fish caught were euthanized with benzocaine just after capture, fixed in 10% formalin solution for 72 hours and then preserved in 70% ethanol. In the lab, specimens were identified to species level, individually measured, and weighed on a semi-analytical balance (accuracy of 1 mg, Ohaus Corporation, Pine Brook, NJ, USA). All necessary permits were obtained for the described study, which complied with all relevant regulations (collection permit number 17559–2; Instituto Chico Mendes de Conservação da Biodiversidade—ICMBio).

### Laboratory experiment

To isolate the effects of temperature on fish body size, we conducted a laboratory experiment exposing a native species to temperatures corresponding the mean minus one standard deviation of the measures taken in forest streams at the coolest time of day (24 °C), and the mean plus one standard deviation of the measures taken in deforested streams at the warmest time of day (32 °C). *Melanorivulus zygonectes* (Cyprinodontiformes, Rivulidae) was selected as our model species for being small, experimentally tractable, and the dominant fish species in headwater streams in the region (accounting for 54% of all individuals collected) [34]. This species occurs almost exclusively in the very shallow margins of water bodies [34; 35], in microhabitats where actual patterns of exposure to high temperatures are surely higher than those we measured in the stream channel. Therefore, 32 °C is likely a conservative estimation of the high temperatures experienced by *M*. *zygonectes* in deforested streams.

In November 2013 we collected ~40 individuals in each of the six streams sampled at Tanguro Ranch, and transported them to the laboratory at the University of São Paulo. Survival during handling and transportation exceeded 90%. Fishes were kept at room temperature in 70 X 40 X 50 cm (width X length X depth) aquariums filled with filtered water to a depth of 20 cm (density of ~0.7 individual.L-1) and fed *ad libitum* with ground TetraMin Fish Flakes (TetraMin, Melle, Germany; 47% protein) until the beginning of the experiment in 18-February-2014 (~90 days). We conducted a fully factorial experiment crossing population of origin (n = 6) and temperature (nominal temperatures 24 and 32 °C), with ten replicates per treatment combination (see the scheme shown on the S2 Fig for additional details on the experimental design). To a large extent this study involves non-invasive techniques, and it was carried out in strict accordance with the national guidelines for the care and use of animals (Conselho Nacional de Controle da Experimentação Animal—CONCEA). Our protocols were approved by the Ethics Committee of the Biosciences Institute for small aquatic organisms (269/2016, process 16.1.574.41, originally requested for work with anuran larvae).

The experimental setup consisted of two water baths 170 X 60 X 10 cm placed in a climatic chamber set at 17 °C, and containing a thermostat to raise the water temperature and a HOBO data logger recording actual temperatures every 30 minutes. Actual temperatures during the experiment were 24.1 ± 0.9 and 32.2 ± 2.3 °C (mean ± standard deviation). Each water bath housed three 50 X 40 X 10 cm aquariums divided in twenty 10 X 10 X 10 cm individual cells (therefore, each water bath housed sixty 1L cells). Water in the water bath continuously circulated around and underneath all three aquaria by means of a submersible pump (S2 Fig).

We selected 20 individuals from each of the six sampled populations aiming to reduce as much as possible interpopulation variation in mean body mass. Mean ± 1 standard deviation (range) mass of selected individuals from each population were APPM: 168.7 ± 53.28 mg (80–273mg); APP2: 141 mg ± 35.72 (94–201 mg); APP2A: 143.95 ± 32.47 mg (80–191 mg); TAN1: 139.85 ± 43.71 mg (94–248 mg); TAN2: 141.22 ± 39.83 mg (82–217 mg); TAN3: 118.25 ± 31.75 mg (88–217mg). Selected fish were randomly placed in ten individual cells at each of the two manipulated temperatures (n = 60 individuals per temperature treatment). Three times a week, the fish were fed ground TetraMin Fish Flakes on a 15 mg per capita daily ration. This feeding regime always exceeded the daily consumption rates for all experimental groups, and at the same time minimized remnants of food which could impair water quality. In these occasions, mortality was registered if it occurred and dead fish were removed. Water in the aquariums was changed weekly. The experiment lasted 60 days, and by the end all survived fishes were weighed individually. We used individual growth (final mass–initial mass) as main response variable, and for quality control purposes, potential reductions in survival over time were also assessed.

### Data analysis

#### Stream temperature and habitat characterization

Water temperature measurements were grouped by land use type regardless of stream, sampling date or time, and difference in mean values were tested with a non-parametric Mann-Whitney test using SigmaPlot 11.0 software (Systat Software, Inc.). Differences among forested and deforested streams in each of the ten environmental characteristics we measured were tested by permutational multivariate analyses of variance (PerMANOVA) with a Monte Carlo permutation procedure (10,000 replicates) done using the PRIMER-E software [36]. These analyses were run in univariate design, included a random factor (“stream”) to account for the variation among multiple samples within each stream, and were based on Euclidean distances of normalized data.

#### Stream fish sampling

To test whether there were body mass differences of fish communities between land uses, we used linear mixed effects models. First, we tested several different random effects structures to account for multiple samples from individual streams and multiple observations for each species. These included 1) a random intercept on stream, 2) random intercept on stream and species, 3) random intercept on stream, random intercept on species, and random slope on species. Model selection among these models using AIC_c_ showed that the third option best captured the random variation in the data (S1 Table). This result, including a random slope on the random “species” effect, demonstrated that not all species responded in the same manner to land use. Using this random effect structure, we then tested whether land use had an effect on body size by comparing models with a fixed effect of “land use” and one with only an intercept. Body mass was log transformed in order to conform to assumptions of normality. We compared these models using AIC_c_ and likelihood ratio tests to evaluate the effect of land use on fish body size. A lower AIC_c_ score signifies a better model. Models that differ by at least two units of AIC_c_ score, denoted by ΔAIC_c_, are considered to differ in their explanatory power, and a difference of 10 signifies a substantially better model [37].

The above models included all species (n = 29) collected across all streams and land use types. The random effects structure suggested that the effect of land use on body size varied across species, however. Therefore, we performed an additional analysis to evaluate variation at the population level. We asked first, if individual species varied in body size between land uses, and, second, how variable these responses were. To do so, we restricted our comparisons to six species for which we collected at least ten individuals in each land use type (*Melanorivulus zygonectes*, *Hyphessobrycon mutabilis*, *Pyrrhulina australis*, *Aequidens michaeli*, *Moenkhausia phaenota*, and *Hyphessobrycon loweae*). These species composed more than 90% of the total fish abundance of this study, so incorporated a vast portion of the fish communities. Here we again used mixed effects models, although independently for each species. These models included a random effect on the intercept to account for multiple observations of each species within a given stream. To evaluate the significance of land use on fish body size for each species, we again compared models with a fixed effect of land use to those with only an intercept using AIC_c_ and likelihood ratio tests.

In addition, we wanted to evaluate whether species composition (e.g., a shift to a community dominated by smaller-bodied species) and/or environmental changes associated with land use (e.g., temperature) explained observed shifts in fish body size. We used two approaches to get at this question with the field study data. First, we used an Analysis of Similarity (ANOSIM) with the Bray-Curtis similarity index and a permutation procedure (10,000 replicates) to test whether the relative abundance of species differed significantly between land uses. We paired this examination with a Similarity Percentage Analysis (SIMPER) to determine which taxa were primarily responsible for observed differences between groups [38]. These tests were run on PAST software [39]. Second, we investigated which specific environmental factors associated with land use change drove these observed shifts in body mass. To do this, we compared models that included environmental factors that differed significantly between land uses with random effects of ‘species’ and ‘stream’ on the intercept. We used this random structure to account for multiple observations within streams and species. We did not test alternative random structures for these models. We standardized environmental variables using Z-scores in order to account for the differences in scale among variables and to be able to directly compare the magnitude of regression coefficients. We again used model selection to compare among models.

#### Laboratory experiment

To test the effects of temperature on fish growth in the laboratory experiment we also used linear mixed effects models and the model selection approach through the Akaike selection criterion (AIC_c_). Initially, four models with the same fixed effect structure, but different random structures were formulated to determine the most appropriate way to include “stream of origin” as a random variable in the final models. We tested the following random effects structures: an effect of origin on the intercept, an effect of origin influencing the effect of temperature on growth, an effect of origin influencing the effect of land use form on growth, and an effect of origin influencing the effect of land use on growth plus an effect of origin influencing the effect of temperature on growth. This analysis indicated that stream of origin should be included in the model as a random factor influencing only the intercept (S2 Table). Once we defined the random structure of the model, six general models were formulated to select the fixed factors. Fixed effect structure described the fish growth as a function of the initial mass, land use in the stream of origin, temperature of rearing, and the interaction between land use and temperature. All analyses of mixed models described in this study were performed using the package “lme4” [40] in R [41], and R^2^ values for the fixed component (R^2^_m_) and complete model (R^2^_c_) were reported according to Nakagawa and Schielzeth [42].

Finally, to analyze fish survival during the lab experiment, we performed a Kaplan-Meier survival analysis with a Log-rank general significance test, and a Holm-Sidak *post-hoc* test for comparisons between groups. We compared the four experimental groups (fishes from forest streams which were reared at 24 °C, fishes from forest streams which were reared at 32 °C, fishes from deforested streams which were reared at 24 °C, and fishes from deforested streams which were reared at 32 °C) disregarding the stream-specific populations. This analysis was done using SigmaPlot 11.0 software (Systat Software, Inc.).

## Results

### Stream water temperature and general habitat characterization

**Fig 1:**
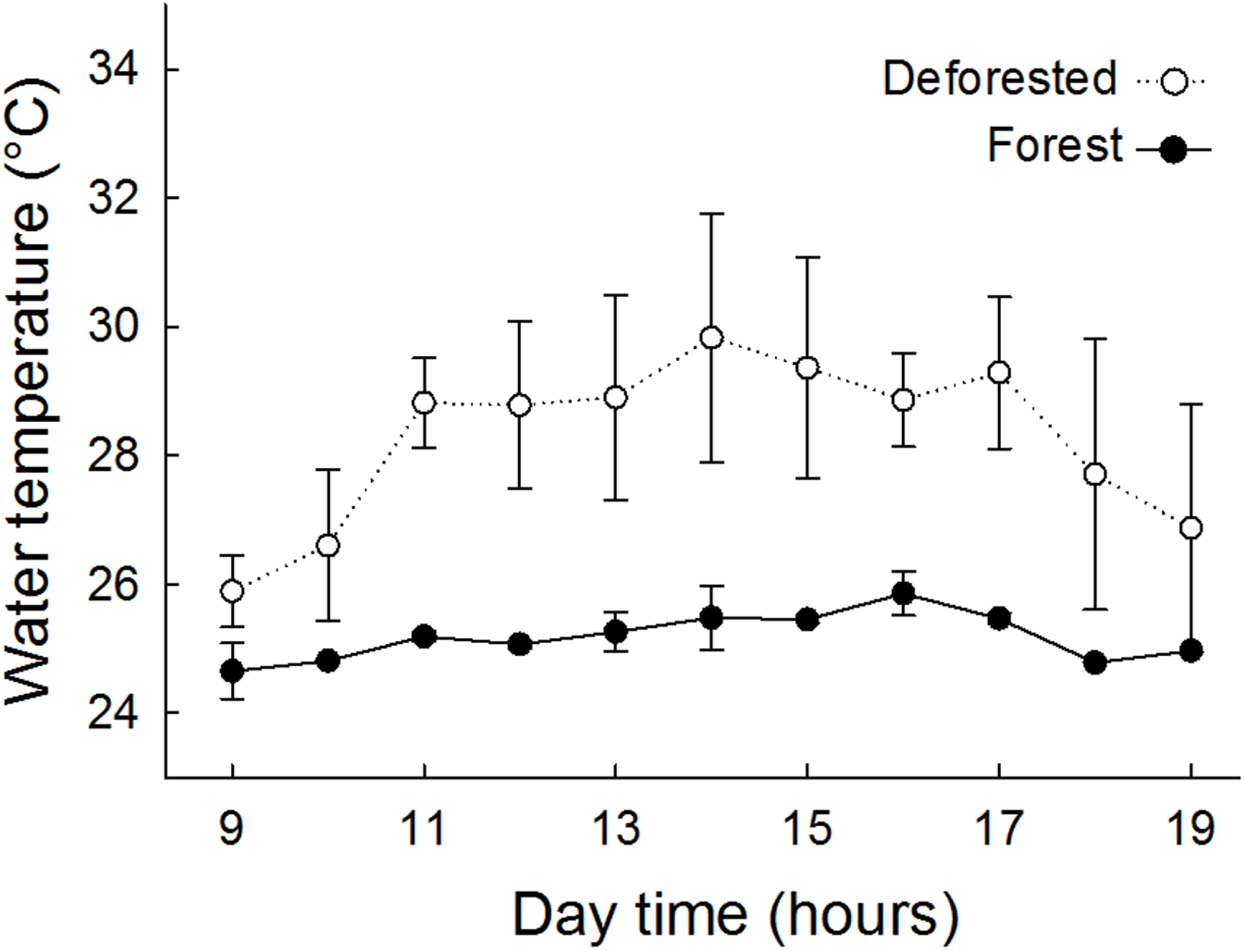
Streams water temperatures. Water temperature throughout the day (between 9 AM and 19 PM hours) in forest (filled circles and solid line) and deforested streams (open circles and dotted line). Symbols represent the mean ± standard deviation of the measures taken in each land use.

Mean water temperature in deforested streams (28.5 °C) was 3.5 °C higher and markedly more variable (range 24.2–34.0 °C, coefficient of variation 7.3%) than in forest streams (average 25.0 °C, range 24.1–26.9 °C, coefficient of variation 2.5%) (Mann-Whitney test, U = 331.5, p <0.001). During the warmest hours of the day (from 13 to 17 hours, for example), mean water temperature in deforested streams was up to 6 °C higher than in forest streams (Fig 1), and the maximum temperatures were nearly 7 °C higher in deforested (34.0 °C) than in forest streams (26.9 °C). Although the temperature data set on which these analyses are based is very small (water temperature measurements were conducted only during the daytime and spanned a temporal range of only a week of a single season), the thermal pattern we found is consistent with the annual and daily patterns shown by Macedo et al. [12] for streams across land uses at Tanguro and across the region.

In addition to higher water temperatures, deforested streams also tended to be deeper, have higher water conductivity, and very low canopy cover (10% on average, against 86% on average in forest streams), which directly affected stream habitat characteristics (as measured as the distribution of stream substrate types). The mean percent cover of coarse benthic organic matter (CBOM) declined from 51% in forest streams to 11% in deforested streams, while the mean percent cover of grasses and macrophytes increased from zero in forest streams to 49% and 14%, respectively, in deforested streams (Table 1).

**Table 1:** General habitat characterization.

Variables	Forest	Deforested	F_1,17_	p value (MC)
Conductivity (μS/cm)	5.3 ± 0.6	18 ± 3.4	154.710	<0.001
pH	5.64 ± 0.2	5.97 ± 0.3	0.157	0.713
Width (m)	1.8 ± 0.3	2.06 ± 0.3	0.156	0.710
Depth (cm)	27 ± 6	45 ± 8	7.038	0.056
Canopy cover (%)	86 ± 2	21 ± 7	113.060	<0.001
Sand (%)	7 ± 4	0 ± 0	4.000	0.117
FBOM (%)	40 ± 6	33 ± 5	1.998	0.226
CBOM (%)	51 ± 6	11 ± 7	1.998	0.004
Grasses (%)	0 ± 0	38 ± 7	15.302	0.004
Macrophytes (%)	0 ± 0	14 ± 6	37.069	0.018

Values of mean ± standard error of the variables measured in the general habitat characterization of forest and deforested stream sites, and results of PerMANOVA tests. FBOM, fine benthic organic matter; CBOM, coarse benthic organic matter.

### Stream fish sampling

**Fig 2:**
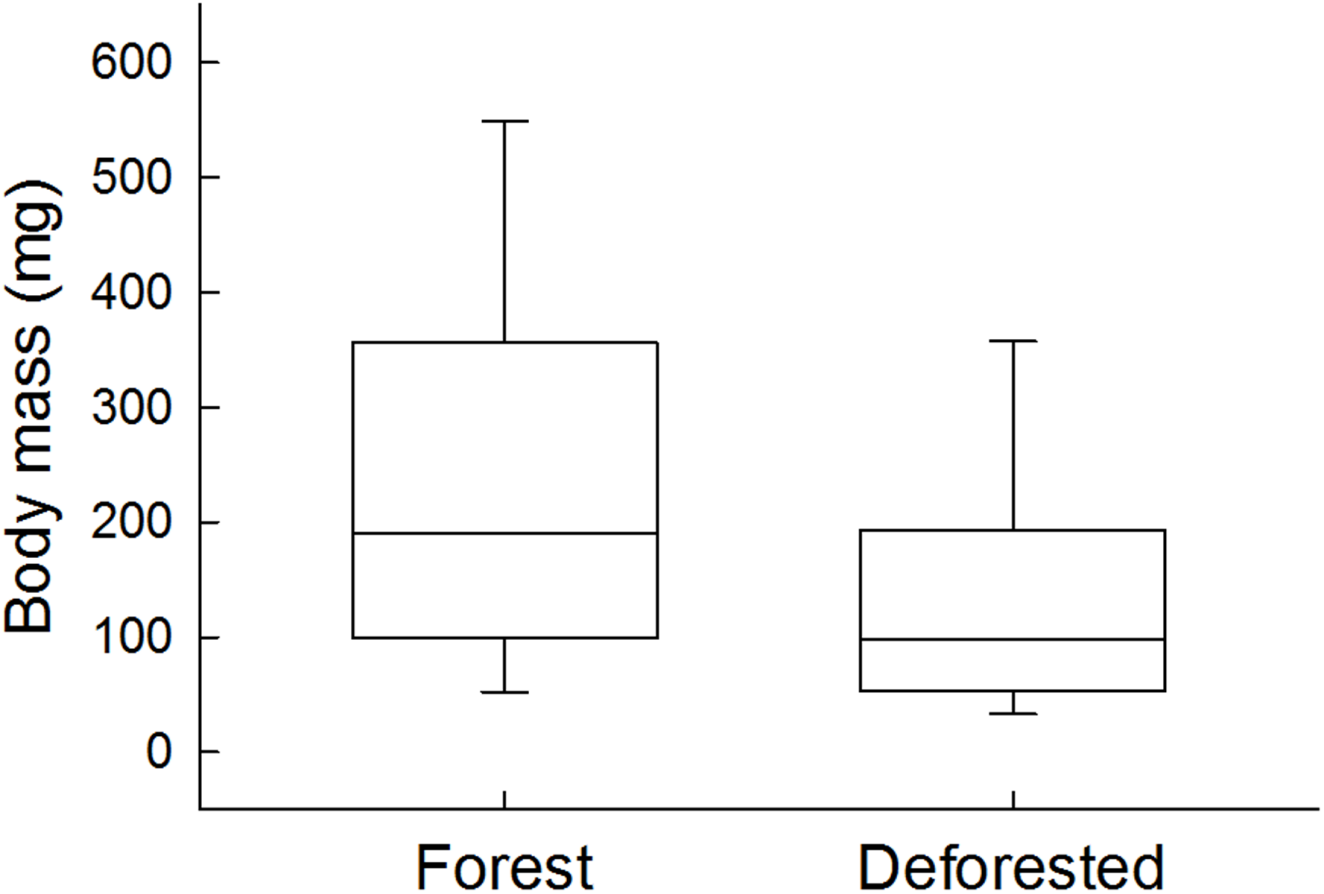
Fish body size at the assemblage level. Fish body mass in assemblages from forest and deforested streams. Boxes represent the first, second (median) and third quartiles, and the whiskers represent the 10th and 90th percentiles of recorded values.

We collected a total of 2504 specimens of 29 species (representing 17 families and 6 orders), of which 801 specimens of 20 species (representing 12 families and 6 orders) were collected in forest streams, and 1703 specimens of 25 species (representing 15 families and 5 orders) were collected in deforested streams. Mean fish body mass in assemblages from deforested streams was 36% smaller than in assemblages from forest streams (Fig 2; Table 2). In deforested streams individual mass was 265±23 mg (mean±s.e.m.), while in the forest streams individual mass was 413±36 mg.

**Table 2:** Fish body size at the assemblage and population levels.

Group	Forest	Deforested	AIC_c_ (~1)	AIC_c_ (~landuse)	ΔAIC_c_	R^2^_m_	R^2^_c_
Assemblage	413 ± 36	265 ± 23	6089	**6087**	2	0.03	0.7
*Melanorivulus zygonectes*	241 ± 8	129 ± 3	3042	**3033**	11	0.15	0.16
*Hyphessobrycon mutabilis*	110 ± 5	62 ± 3	399	**396**	3	0.19	0.3
*Pyrrhulina australis*	313 ± 59	226 ± 11	**539**	541	2	0.001	0.2
*Aequidens michaeli*	350 ± 98	1014 ± 218	696	696	0	0.01	0.5
*Moenkhausia phaeonota*	512 ± 41	258 ± 22	356	**353**	5	0.15	0.17
*Hyphessobrycon loweae*	293 ± 13	130 ± 18	94	**92**	2	0.26	0.4

Values of mean body mass ± standard error of fish assemblages and populations from forest and deforested streams, and respective AIC_c_ model results. ~1 indicates that the model contained only an intercept and ~landuse means that the model included landuse as a fixed effect. Number of parameters (*k*) for models ~1 and ~landuse were 3 and 4, respectively. Models with smaller AIC_c_ values and considered different via ΔAIC_c_ are in bold. The R^2^_m_ and R^2^_c_ values are reported for the better models. AIC_c_, corrected Akaike Information Criteria; ΔAIC_c_, difference in AIC_c_ between ~1 and ~landuse models; R^2^_m_, R^2^ value for the fixed component; R^2^_c_, R^2^ value for the complete model.

**Fig 3:**
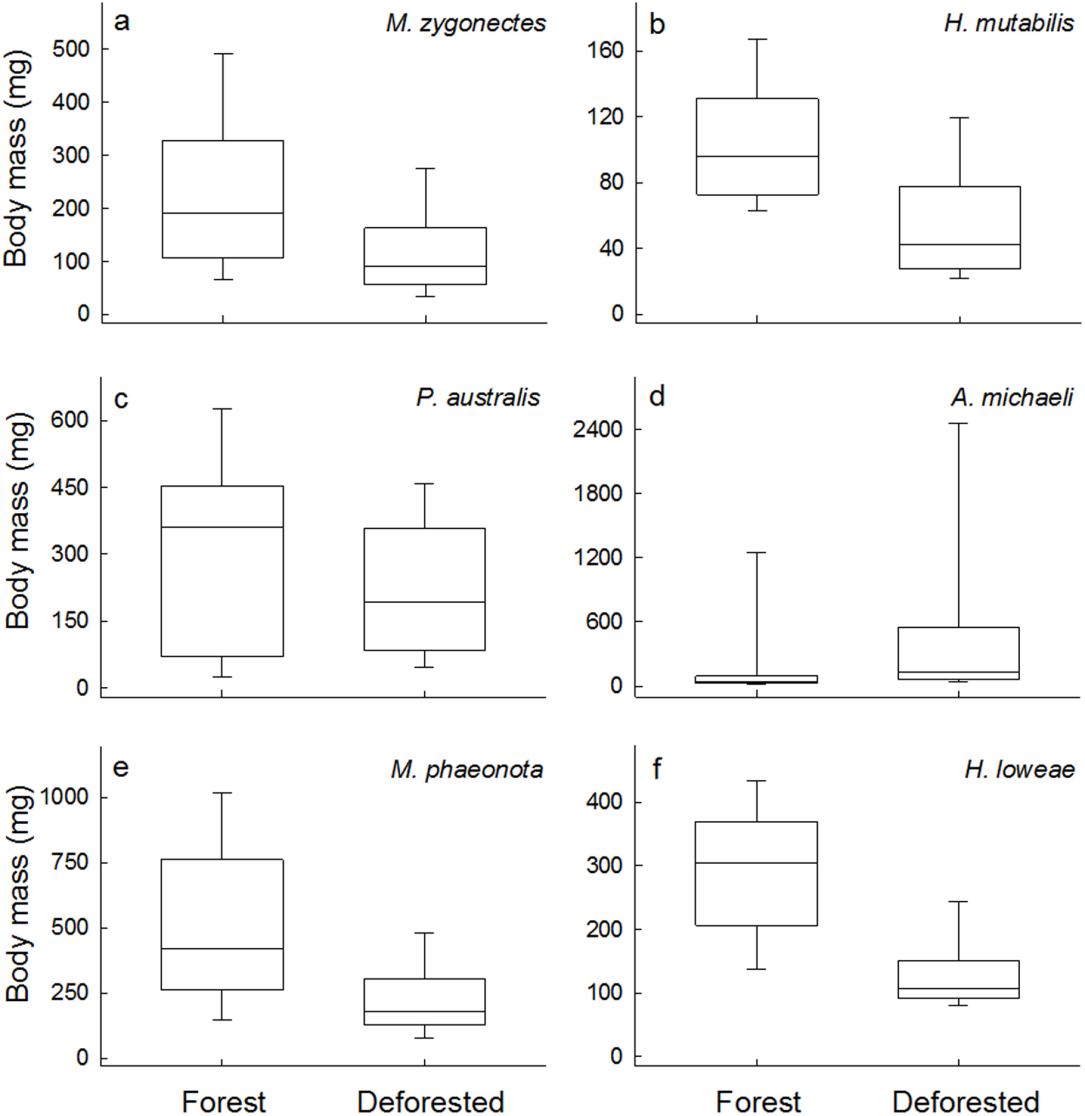
Fish body size at the population level. Body mass of fishes from populations of forest and deforested streams, to the species a) *Melanorivulus zygonectes*, b) *Hyphessobrycon mutabilis*, c) *Pyrrhulina australis*, d) *Aequidens michaeli*, e) *Moenkhausia phaenota*, and f) *Hyphessobrycon loweae*. Boxes represent the first, second (median) and third quartiles, and the whiskers represent the 10th and 90th percentiles of recorded values.

Among the six most abundant species, four had lower mean body mass in populations from deforested streams relative to populations from forest streams. The species *Melanorivulus zygonectes*, *Hyphessobrycon mutabilis*, *Moenkhausia phaeonota*, and *Hyphessobrycon loweae*, had mean individual mass 46%, 43%, 49% and 55% lower in populations from deforested streams relative to populations from forest streams (Fig 3; Table 2), respectively, and models with “land use” as a factor had lower AIC_c_ values (Table 2). Mean body mass of *Pyrrhulina australis* was lower (about 27%) in deforested streams, but did not differ significantly by land use (Fig 3c; Table 2). The only species that had larger mean body mass in deforested streams was *Aequidens michaeli*, which was on average three times larger than in forest populations (Fig 3d; Table 2).

Another factor that could influence average body mass is a turnover in community composition toward more small-bodied species. We found that the relative abundance of fish species differed significantly between land uses (ANOSIM *p =* 0.02), and that the taxa primarily responsible for the observed difference was *M*. *zygonectes* (similarity percentage analysis, SIMPER). This species is one of the smallest in our dataset, and its increased mean relative abundance in deforested streams (from 46% in forest streams to 54% in deforested streams) accounted for 27% of the total dissimilarity between groups. The six most abundant species together accounted for nearly 82% of the total dissimilarity among land uses, while all 22 remaining species accounted for the remaining 18%.

The results from our models testing the effect of land use on the assemblage and population levels showed that body size differed between land uses in both cases. We also tested which specific environmental factors associated with land use change affected body size. The model that explained the most variation in the data (highest R^2^_m_) and that had the lowest AIC_c_ included temperature, canopy cover, coarse benthic organic matter (CBOM), and conductivity as main effects (Table 3). Model coefficients (S3 Table) indicated that temperature (coefficient = -0.58), conductivity (coefficient = -0.66), and coarse benthic organic matter (coefficient = -0.52) had negative effects on body size, while canopy cover had a positive effect (coefficient = 0.7). All factors included in the final model were significant via likelihood ratio tests (p<0.001).

**Table 3:** Effects of environmental factors on fish body mass.

Model formula	k	AIC_c_	ΔAIC_c_	R^2^_m_	R^2^_c_
Mass ~ temp + cond + cbom + canopy	7	4284.2	0.0	0.28	0.74
Mass ~ temp + cond + cbom + canopy + grass	8	4290.2	6.0	0.28	0.83
Mass ~ temp + cond + cbom	6	4291.8	7.6	0.13	0.72
Mass ~ temp + cond + cbom + canopy + grass + depth	9	4292.9	8.7	0.16	0.82
Mass ~ cond + cbom + canopy	6	4298.6	14.4	0.08	0.71
Mass ~ temp + cond	5	4326.6	42.5	0.1	0.71
Mass ~ cond + cbom	5	4329.2	45.1	0.02	0.72
Mass ~ temp + cond + canopy	6	4330.4	46.2	0.12	0.71
Mass ~ cond + canopy	5	4333.1	49.0	0.07	0.7
Mass ~ cbom	4	4336.4	52.3	0.02	0.73
Mass ~ canopy	4	4337.4	53.3	0.06	0.69
Mass ~ cond	4	4339.0	54.9	0.006	0.69
Mass ~ temp + canopy	5	4339.2	55.1	0.06	0.69
Mass ~ temp + cbom	5	4340.6	56.5	0.03	0.74
Mass ~ 1	3	4345.1	60.9	0	0.69
Mass ~ temp	4	4348.7	64.6	0.002	0.7

Results of models evaluating the relative importance of environmental factors with regard the observed shifts in body mass. The best models were considered those with lowest AIC_c_ score. All models had n = 1685. Temp, temperature; cond, conductivity; cbom, coarse benthic organic matter; K, number of parameters in the model; AIC_c_, corrected Akaike Information Criteria; ΔAIC_c_, difference in AIC_c_ between current and better model; R^2^_m_, R^2^ value for the fixed component; R^2^_c_, R^2^ value for the complete model.

### Laboratory experiment

**Fig 4:**
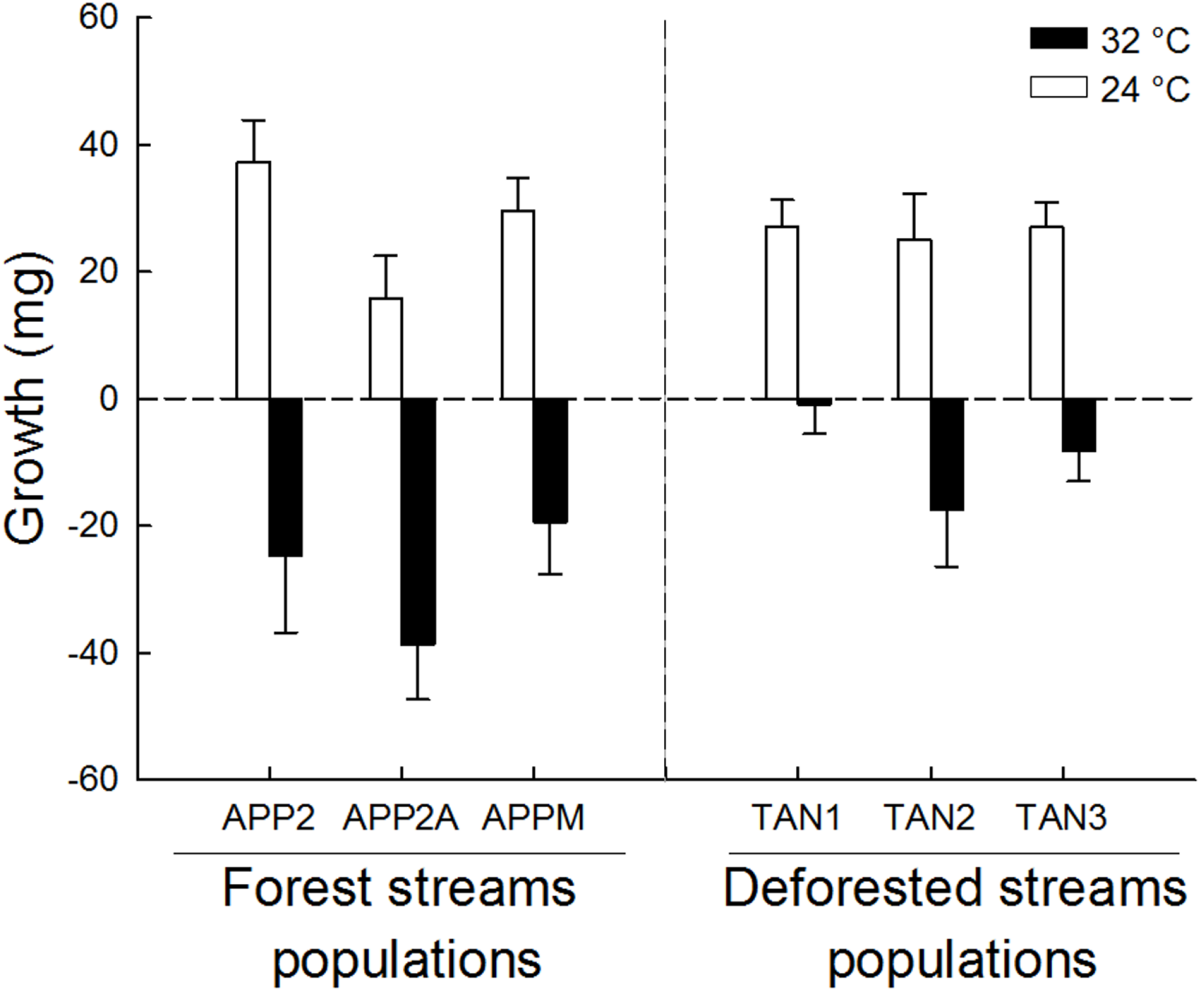
Fish growth in the lab experiment. Mass gain of *Melanorivulus zygonectes* from populations of forest and deforested streams reared over 60 days at 24 °C (white bars) and 32 °C (dark bars). Bars represent mean ± 1 standard error of 10 individuals.

Growth of *M*. *zygonectes* was negatively affected by higher temperatures, especially in populations from forest streams. On average, individuals from forest populations reared at 24 °C gained 27.5 mg and those reared at 32 °C lost 27.5 mg during the experiment, while individuals from deforested streams populations reared at 24 °C gained 26.4 mg and those reared at 32 °C lost just 9.5 mg (Fig 4). The model that best explained these patterns (Table 4) had an interaction factor between population origin and temperature, suggesting that the origin of the populations affected the growth response to temperature, and that populations from deforested streams had lower sensitivity to warming. This pattern also applied to the survival of *M*. *zygonectes* (LogRank Statistic = 12,492, df = 3, *p* = 0.006; see S3 Fig for survival curves graph). By the end of the experiment, mean survivorship of populations from deforested streams was 90% at both 24°C and 32 °C; whereas in forest populations reared at 24°C was 93%, and dropped to 66.6% at 32 °C (see S4 Table for results of Holm-Sidak *post-hoc* test for comparisons between groups).

**Table 4:** Factors affecting fish growth in the lab experiment.

Model formula	k	AIC_c_	ΔAIC_c_	R^2^_m_	R^2^_c_
Growth ~ mass0 + landuse + temp + landuse: temp + (1|origin)	7	871.4	0.0	0.59	0.66
Growth ~ landuse + temp + landuse: temp + (1|origin)	6	877.2	5.8	0.56	0.60
Growth ~ mass0 + landuse + temp + (1|origin)	6	882.1	10.7	0.57	0.63
Growth ~ mass0 + landuse + (1|origin)	5	971.3	99.9	0.04	0.06
Growth ~ mass0 + temp + (1|origin)	5	885.8	14.4	0.58	0.63
Growth ~ mass0 + (1|origin)	4	974.7	103.3	0.04	0.04
Growth ~ (1| origin)	3	973.6	102.2	0.00	0.00

Results of models evaluating the relative importance of factors affecting growth of *Melanorivulus zygonectes* in the lab experiment. The best models were considered those with lowest AIC_c_ score. All models had n = 101. Mass0, initial mass of experimental individuals; temp, temperature; K, number of parameters in the model; AIC_c_, corrected Akaike Information Criteria; ΔAIC_c_, difference in AIC_c_ between current and better model; R^2^_m_, R^2^ value for the fixed component; R^2^_c_, R^2^ value for the complete model.

## Discussion

Our results support the hypothesis that deforestation and the resulting increase in stream temperatures in a rapidly developing region of southern Amazonia are important factors influencing fish body size at the assemblage, population, and individual levels. While our field results show that other factors associated with deforestation in addition to temperature, such as canopy openness and changes in organic matter inputs also influence fish body size, our experimental results demonstrate that temperature alone has a substantial effect on the growth and size of fish from this region. The findings of our study have important implications for conservation of fish diversity in response to changing climate and land use. Body size influences virtually every aspect of organism’s biology [20; 21], and Amazonian headwaters tend to harbor rich, endemic, and comparatively unknown fish faunas. Freshwater fish play a key functional role in aquatic food webs, and may serve as important natural, cultural, and recreational resources [43]. If future studies demonstrate similar effects in larger fishes from higher order streams, warming could have important consequences for ecosystem services (e.g., food, income, nutrition) to humans.

Several observational and experimental studies have shown that increases in temperature can reduce body size of fish and other ectothermic animals (e.g., [18; 22; 23; 44; 45; 46; 47; 48]). However, the vast majority of these studies have been done with marine and/or temperate species, while tropical regions harbor the most freshwater fish species in the world [49], and which may have narrow thermal tolerances and therefore be vulnerable to even modest warming [50]. To the best of our knowledge, the only study in the literature relating temperature and body size in freshwater tropical fishes was the unpublished study of G. Lacerot et al., which demonstrated that in lakes along a latitudinal gradient in South America (from southern Argentina to northeastern Brazil) mean fish body size is positively correlated with latitude or, in other words, mean fish size is smaller in lakes located in regions with higher mean annual temperatures [51]. The same latitudinal pattern of size variation was observed in freshwater fish assemblages in Europe [51; 52] and, for example, in European and North American populations of the arctic char (*Salvelinus alpines*) [53; 54], but in freshwater species of North America this pattern is not as clear and the opposite trend has been observed for many species [55].

The 36% reduction in fish body size we observed at assemblage level in deforested streams can be partly attributed to the 8% increase in mean relative abundance of a small-bodied species (namely *M*. *zygonectes*). This increase in abundance was related to a higher availability of shallow areas, the preferred habitat of *M*. *zygonectes*, in riparian floodplains of deforested streams (Schiesari et al. unpublished). However, in addition to making up a larger proportion of the assemblage, individuals of this species were also significantly smaller in deforested streams. Actually, among the six most abundant species in our study area, four (including *M*. *zygonectes*) had mean body sizes that were smaller (43–55%) in populations from deforested streams. Thus, body size reductions at population level in these streams were the most likely driving factor underlying the body size reductions observed at assemblage level. Our model results support this assessment: we found that a model with a random effect of “species” on the slope described the patterns in fish body size better than one not accounting for differential species effects (S1 Table). Such reductions in fish body size were considerably larger than the documented and projected effects of climate change on fish size found in the literature (11–24%) [44; 47]. This is not surprising, however, given that the temperature increase we recorded in deforested streams was substantially higher than that expected to result solely from climate change [13], and because other factors likely contributed to the body size reductions observed in the field study. Deforested streams were on average 3.5 °C warmer than forest streams, but up to 6 °C warmer during the middle of the day.

The only species whose populations from deforested streams had mean body size larger than forest populations was *Aequidens michaeli*. Although larger body sizes at higher temperatures seem to be an exception to the general biological pattern, it has been described for some organisms, like species living in the limit of their geographic distribution [56; 57] or in seasonal or altered environments [58; 59; 60]. It is possible, therefore, that the exception found for *A*. *michaeli* might be found for other species not analyzed by this study. For *A*. *michaeli*, in particular, it is also possible that mean body size was higher in deforested streams because such environments are more favorable for them due to other factors. This species appears to benefit from stream impoundment and reservoirs, which were present in all deforested streams (but not in forest streams) and could be a source of large-sized individuals to lotic stream reaches [34].

A reduced mean body size at the population level might be a result of an increase in the proportion of young individuals and/or a decrease in individuals’ body size relative to age [18]. However, we are not able to say how much these factors affected our results because we did not measure fish age. In addition, the body size differences we observed in our field study could also have been affected by factors such as differences in individual size at birth [61], differences in growth period length [62], genetic differences in individual growth rates [63], increased and/or size-selective predation [24; 64; 65], or differences in food quantity and/or quality available in each environment [17; 63; 66]. Food constraints reduce growth, and any environmental conditions that reduce growth adversely affect organism body size [66].

In our study, the inclusion of both canopy cover and coarse benthic organic matter (CBOM) in the final models suggests that, in addition to temperature, alterations in resource availability could also be important for explaining the changes in fish body size observed in the field. Average canopy cover and CBOM declined from 86% and 51% in forest streams to 10% and 11% in deforested streams, respectively. The inputs of organic matter derived from adjacent forests are one of the main sources of carbon to the food web in forested headwater streams, and consequently, deforestation and lower organic matter inputs can reduce fish body size [67]. It is possible that fishes whose diets rely mostly on organic materials provided by riparian forests (seeds, fruits, leaves, terrestrial invertebrates) had faced low food availability in open canopy deforested streams. However, the lower availability of CBOM does not explain the body size reductions we observed in deforested streams, since this variable had a negative effect on fish body size (S3 Table). Our field study and model results showed that temperature negatively impacted fish body size in a natural setting and the lab experiment demonstrated reduced body size and survival at high temperature in the absence of other factors.

Other studies have suggested that even water temperatures below what we observed in the field might be detrimental to other Brazilian fish. For example, 28 °C was found to impair the growth of *Austrolebias wolterstorffi* [68] and 25 °C reduced the body condition factor of *Austrolebias nigrofasciatus* [69]. The mass loss of *M*. *zygonectes* at 32 °C shows that temperatures similar to those occurring in the warmer hours of the day in deforested stream channels are sufficient to cause growth inhibition and reduce body size in fishes, even in species that seem to be relatively tolerant to warming, such as *M*. *zygonectes*. A recent review [23] investigating the effects of warming on the body size of organisms from different taxonomic groups (including bony fish, amphibians, cnidarians, crustaceans, and several orders of insects) demonstrated that, for each degree of temperature increase, the body mass of aquatic organisms declines on average 3.5%. In the lab experiment we conducted, the mass of *M*. *zygonectes* was reduced by just 2.6% for each one degree increase. The physiological mechanisms underlying such body size reductions were not assessed in our study, but several processes could have been involved, including increased energy expenditure in body maintenance at high temperature, reduced food intake rates and/or reduction of food assimilation efficiency [23; 70; 71; 72; 73; 74].

We also found compelling evidence for differences in the ability of populations reared under different environmental conditions/land uses to cope with warming in our lab experiment. Fishes originating from forest populations suffered a more pronounced mass loss and survival reduction at 32°C than fishes originating from deforested streams populations. Such different responses between populations which were experiencing similar temperatures in the experiment suggest that warming promote genetic adaptations and/or non-genetic effects (e.g., maternal and developmental epigenetic effects) in deforested stream populations towards a lower sensitivity to warming. Which of these mechanisms underlie the results of our study is unknown, and remains to be investigated in future studies. Understanding the capacity and the means by which organisms may cope with warming is critical to predicting their future persistence under environmental warming driven by climate changes [75; 76], and by deforestation as well.

In conclusion, our study suggests that deforestation-driven stream warming is likely to negatively affect fish body size in tropical stream fish assemblages and support the hypothesis that declining body size is a general biological response to warming. In addition, our study also suggests that other changes in stream conditions driven by deforestation, like canopy openness and changes in organic matter inputs, can affect fish body size. Declining fish size may, in the long term, negatively affect global biodiversity and ecosystem services, including food supplies for nearly a billion people who depend on fish as their primary protein source [46]. In the Amazon, for example, fish consumption is relatively high compared with the global average [77], and our field site lies just upstream from the Xingu Indigenous Park, whose residents depend heavily on fish protein for subsistence [78]. Deforestation has already reached 20% of the Amazon basin area, and therefore it is possible that body size reductions mediated by stream warming are occurring in fishes and other aquatic animals throughout the Arc of Deforestation, where headwater streams collectively account for a large fraction of regional biodiversity. Such warming is likely to interact with future warming induced by climate change, further amplifying potential negative effects of increased temperature on biological systems. However, it is possible that management practices adopted at local and regional level, such as avoiding impoundments, restoring river flow, and conserving and restoring riparian forests, can efficiently mitigate headwater warming and the resulting effects on aquatic biota.

## Supporting information

S1 Table Random effects structure comparisons for assemblage model. Results of models evaluating the random effects structure for assemblage model. The best models were considered those with lowest AIC_c_ score. All models had n = 2426. K, number of parameters in the model; AIC_c_, corrected Akaike Information Criteria; ΔAIC_c_, difference in AIC_c_ between current and better model. Model Random Structures: RISt, Random Intercept on Stream; RISp, Random Intercept on Species; RSSt, Random Slope on Stream; RSSp, Random Slope on Species; RIStSp, Random Intercept on Stream and Species; RISSp, Random Intercept and Slope on Species.
(DOCX)

S2 Table Selection of the random structure of models evaluating fish growth in the lab experiment. Results of models evaluating the most appropriate way to include “stream of origin” as a random variable in the final models of fish growth in the lab experiment. The best models were considered those with lowest AIC_c_ score. All models had n = 101. Origin, stream of origin; Mass0, initial mass of experimental individuals; temp, temperature; K, number of parameters in the model; AIC_c_, corrected Akaike Information Criteria; ΔAIC_c_, difference in AIC_c_ between current and better model. Model Random Structures: RIO, Random Intercept on Origin; RSO, Random Intercept and Slope on Origin; RST, Random Slope on Temperature.
(DOCX)

S3 Table Model coefficients for best environmental model. Estimates and standard errors of the model coefficients for best environmental model. CBOM, coarse benthic organic matter.
(DOCX)

S4 Table Survival comparisons between experimental groups. Statistical results of Holm-Sidak *post-hoc* tests for comparisons between groups for the survival of *Melanorivulus zygonectes* in the laboratory experiment. *represents significant difference (*p* value smaller than critical level).
(DOCX)

S1 Fig Location of study area and fish sampling sites. The inset shows the place of Tanguro Ranch in the southeastern region of the Amazonian Arc of Deforestation, with green representing closed canopy forests, yellow representing deforested areas and uncolored areas representing native savannas. From South to North, streams sampled are APPM, APP2A, APP2, TAN1, TAN2, and TAN3.
(TIF)

S2 Fig Scheme of experimental design and setup. Two water baths 170 x 60 x 10 cm (width x length x depth) were placed in a climatic chamber set at 17°C, and containing a thermostat to raise the water temperature to 24 and 32 °C. Each water bath housed three 50 x 40 x 10 cm aquariums divided in twenty 10 x 10 x 10 cm individual cells (therefore, each water bath housed sixty 1L cells). Water in the water bath continuously circulated around and underneath all three aquaria by means of a submersible pump. Ten individuals from six streams/populations were randomly placed at each of the two manipulated temperatures (n = 60 individuals per temperature treatment).
(TIF)

S3 Fig Fish survival curves in the lab experiment. Survivorship of *Melanorivulus zygonectes* from forest (circles and solid lines) and deforested (triangles and dashed lines) streams populations reared at 24 °C (filled symbols) and 32 °C (blank symbols) during 60 days of experiment. Symbols represent mean values (n = 30 individuals) and error bars represent standard errors.
(TIF)

S1 Data Data set of fish body size. (XLSX)

S2 Data Data set of temperature by day time. (XLSX)

S3 Data Data set of environmental characteristics. (XLSX)

S4 Data Data set of fish growth in the lab experiment. (XLSX)
