# Supplementary material for: Deforestation and stream warming affect body size of Amazonian fishes
Source: PLoS One. 2018 May 2;13(5):e0196560. doi: 10.1371/journal.pone.0196560 (PMC5931656; doi:10.1371/journal.pone.0196560)
Supplement: S1 Table — Results of models evaluating the random effects structure for assemblage model. The best models were considered those with lowest AICc score. All models had n = 2426. K, number of parameters in the model; AICc, corrected Akaike Information Criteria; ΔAICc, difference in AICc between current and better model. Model Random Structures: RISt, Random Intercept on Stream; RISp, Random Intercept on Species; RSSt, Random Slope on Stream; RSSp, Random Slope on Species; RIStSp, Random Intercept on Stream and Species; RISSp, Random Intercept and Slope on Species. (DOCX) [file pone.0196560.s001.docx]

**S1 Table****. Random effects structure comparisons for assemblage model.**

| **Model Formula** | **Model Random Structure** | **k** | **AIC_c_** | **∆AIC_c_** |
| --- | --- | --- | --- | --- |
| Mass ~ Landuse + (Landuse\|Species) + (1\|Stream) | RIStSp, RSSp | 6 | 6087,8 | 0 |
| Mass ~ Landuse + (Landuse\|Species) | RISSp | 5 | 6105,9 | 18,16 |
| Mass ~ Landuse + (1\|Species) + (Landuse\|Stream) | RIStSp, RSSt | 6 | 6190,9 | 103,17 |
| Mass ~ Landuse + (1\|Species) + (1\|Stream) | RIStSp | 5 | 6191,1 | 103,3 |
| Mass ~ Landuse + (1\|Species) | RISp | 4 | 6223,3 | 135,5 |
| Mass ~ Landuse + (1\|Stream) | RISt | 4 | 6943,3 | 855,5 |

Results of models evaluating the random effects structure for assemblage model. The best models were considered those with lowest AIC_c_ score. All models had n=2426. K, number of parameters in the model; AIC_c_, Akaike Information Criteria corrected; ∆AIC_c_, difference in AIC_c_ between current and better model. Model Random Structures: RISt, Random Intercept on Stream; RISp, Random Intercept on Species; RSSt, Random Slope on Stream; RSSp, Random Slope on Species; RIStSp, Random Intercept on Stream and Species; RISSp, Random Intercept and Slope on Species.
