# Supplementary material for: Deforestation and stream warming affect body size of Amazonian fishes
Source: PLoS One. 2018 May 2;13(5):e0196560. doi: 10.1371/journal.pone.0196560 (PMC5931656; doi:10.1371/journal.pone.0196560)
Supplement: S2 Table — Results of models evaluating the most appropriate way to include “stream of origin” as a random variable in the final models of fish growth in the lab experiment. The best models were considered those with lowest AICc score. All models had n = 101. Origin, stream of origin; Mass0, initial mass of experimental individuals; temp, temperature; K, number of parameters in the model; AICc, corrected Akaike Information Criteria; ΔAICc, difference in AICc between current and better model. Model Random Structures: RIO, Random Intercept on Origin; RSO, Random Intercept and Slope on Origin; RST, Random Slope on Temperature. (DOCX) [file pone.0196560.s002.docx]

**S2 Table. Selection of the random structure of models evaluating fish growth in the lab experiment.**

| **Model Formula** | **Model Random Structure** | **k** | **AIC_c_** | **∆AIC_c_** |
| --- | --- | --- | --- | --- |
| Growth ~ mass0 + landuse + temp + landuse:temp + (1\|origin) | RIO | 7 | 871.4 | 0.0 |
| Growth ~ mass0 + landuse + temp + landuse:temp + (landuse\|origin) | RISO | 9 | 874.7 | 3.3 |
| Growth ~ mass0 + landuse + temp + landuse:temp + (temp\|origin) | RST | 9 | 876.1 | 4.7 |
| Growth ~ mass0 + landuse + temp + landuse:temp + (landuse\|origin) + (temp\|origin) | RISO, RST | 12 | 882.2 | 10.8 |

Results of models evaluating the most appropriate way to include “stream of origin” as a random variable in the final models of fish growth in the lab experiment. The best models were considered those with lowest AIC_c_ score. All models had n=101. Origin, stream of origin; Mass0, initial mass of experimental individuals; temp, temperature; K, number of parameters in the model; AIC_c_, Akaike Information Criteria corrected; ∆AIC_c_, difference in AIC_c_ between current and better model. Model Random Structures: RIO, Random Intercept on Origin; RSO, Random Intercept and Slope on Origin; RST, Random Slope on Temperature.
