# Supplementary material for: Deforestation and stream warming affect body size of Amazonian fishes
Source: PLoS One. 2018 May 2;13(5):e0196560. doi: 10.1371/journal.pone.0196560 (PMC5931656; doi:10.1371/journal.pone.0196560)
Supplement: S3 Table — Estimates and standard errors of the model coefficients for best environmental model. CBOM, coarse benthic organic matter. (DOCX) [file pone.0196560.s003.docx]

**S3 Table. Model coefficients for best environmental model.**

| **Factor** | **Estimate** | **Std. Error** |
| --- | --- | --- |
| Intercept | 5,88 | 0,44 |
| Temperature | -0,58 | 0,12 |
| Conductivity | -0,66 | 0,11 |
| CBOM | -0,52 | 0,07 |
| Canopy cover | 0,7 | 0,19 |

Estimates and standard errors of the model coefficients for best environmental model. CBOM, coarse benthic organic matter.
