# Supplementary material for: Deforestation and stream warming affect body size of Amazonian fishes
Source: PLoS One. 2018 May 2;13(5):e0196560. doi: 10.1371/journal.pone.0196560 (PMC5931656; doi:10.1371/journal.pone.0196560)
Supplement: S4 Table — Statistical results of Holm-Sidak post-hoc tests for comparisons between groups for the survival of Melanorivulus zygonectes in the laboratory experiment. *represents significant difference (p value smaller than critical level). (DOCX) [file pone.0196560.s004.docx]

**S4 Table. Survivorship comparisons between experimental groups.**

| **Comparison** | **Statistic** | **p value** | **critical level** |
| --- | --- | --- | --- |
| Forest 24 °C Vs Forest 32 °C * | 6.938 | 0.00844 | 0.00851 |
| Forest 24 °C Vs Deforested 24 °C | 0.264 | 0.608 | 0.0170 |
| Forest 24 °C Vs Deforested 32 °C | 0.181 | 0.671 | 0.0253 |
| Forest 32 °C Vs Deforested d 24 °C | 5.024 | 0.0250 | 0.0127 |
| Forest 32 °C Vs Deforested 32 °C | 5.254 | 0.0219 | 0.0102 |
| Cropland 24 °C Vs Deforested 32 °C | 0.00331 | 0.954 | 0.05 |

Statistical results of Holm-Sidak *post-hoc* tests for comparisons between groups for the survival of *Melanorivulus zygonectes* in the laboratory experiment. *represents significant difference (*p* value smaller than critical level).
