# Supplementary figures and images for: Deforestation and stream warming affect body size of Amazonian fishes
Source: PLoS One. 2018 May 2;13(5):e0196560. doi: 10.1371/journal.pone.0196560 (PMC5931656; doi:10.1371/journal.pone.0196560)

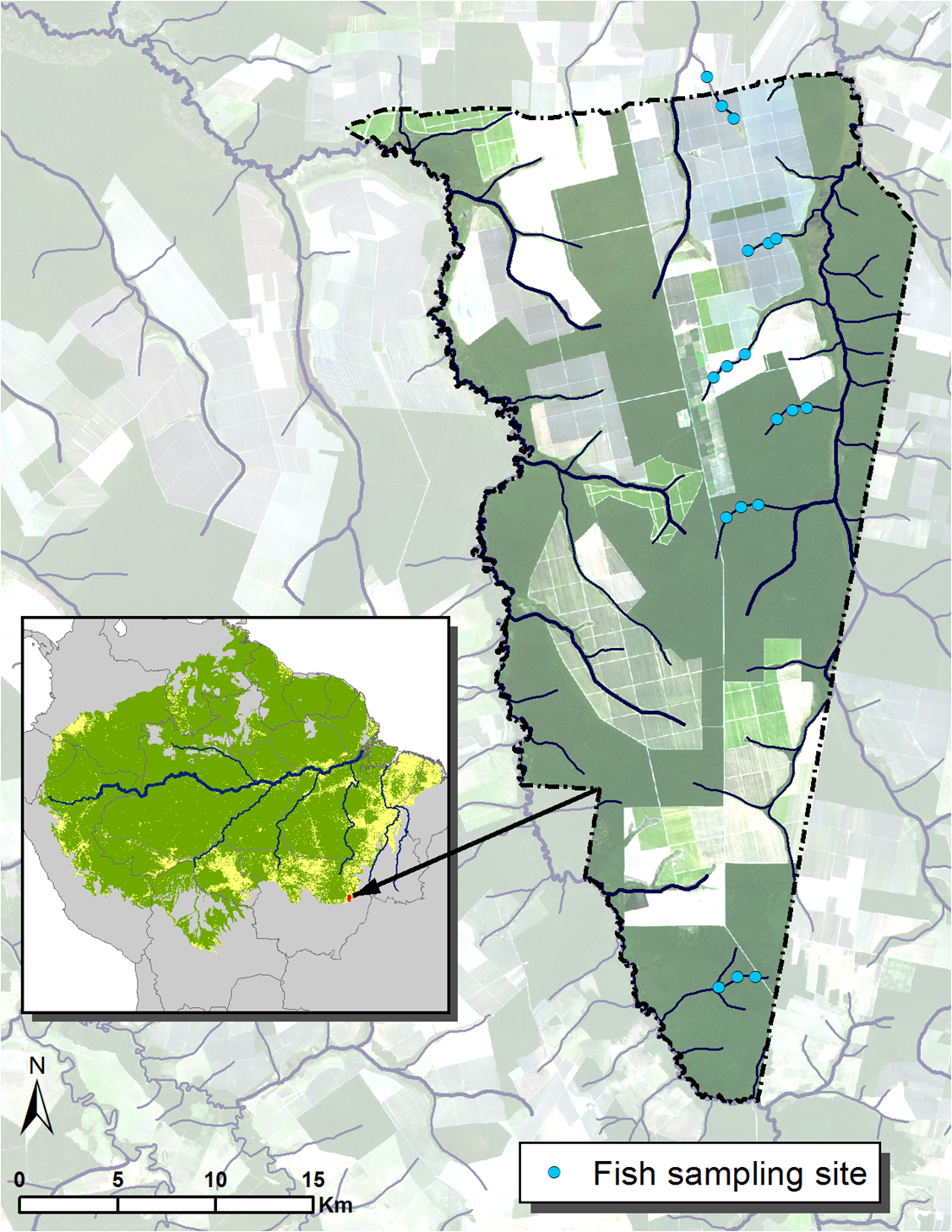

Supplement: S1 Fig — The inset shows the place of Tanguro Ranch in the southeastern region of the Amazonian Arc of Deforestation, with green representing closed canopy forests, yellow representing deforested areas and uncolored areas representing native savannas. From South to North, streams sampled are APPM, APP2A, APP2, TAN1, TAN2, and TAN3. (TIF) [file pone.0196560.s005.tif]

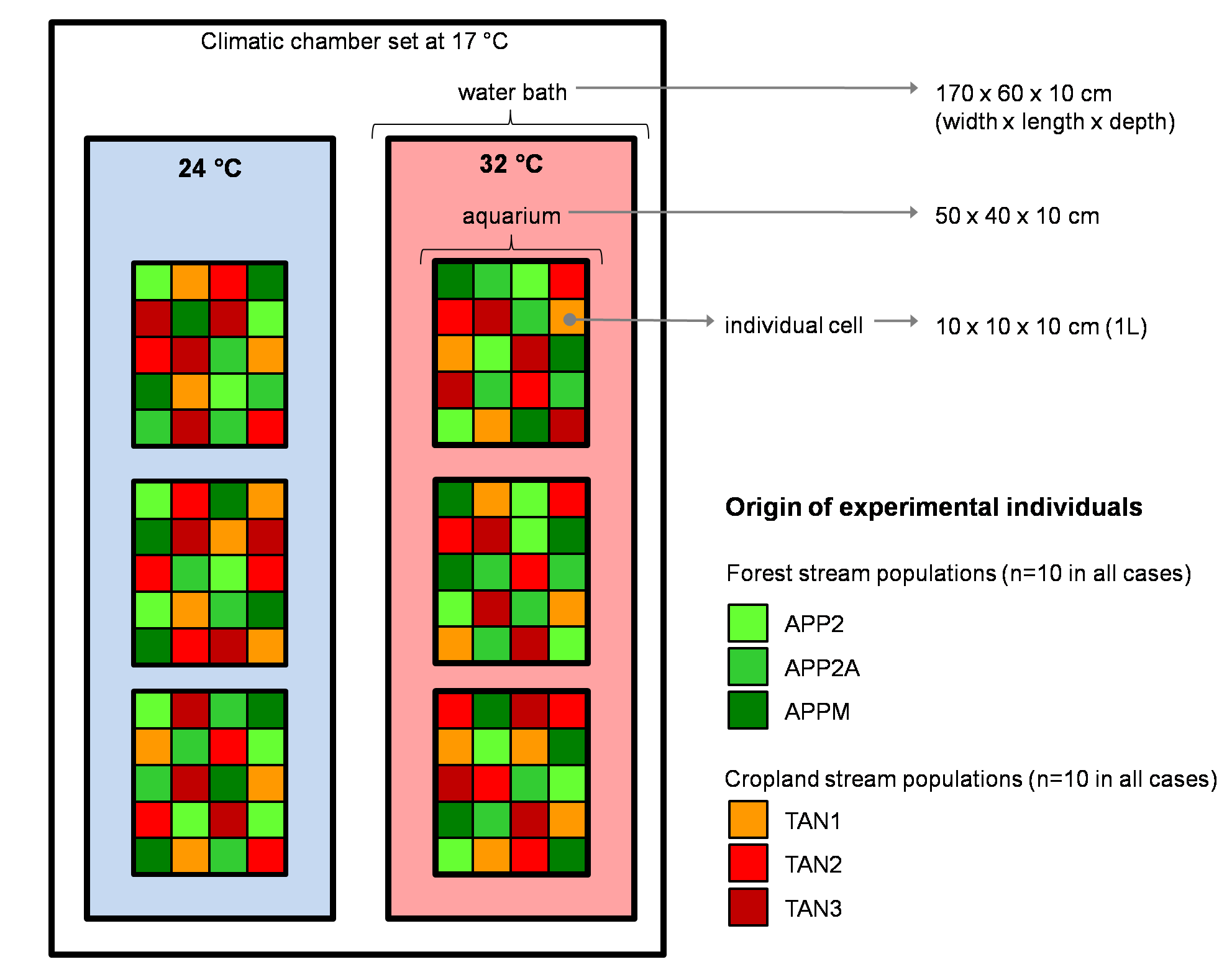

Supplement: S2 Fig — Two water baths 170 x 60 x 10 cm (width x length x depth) were placed in a climatic chamber set at 17°C, and containing a thermostat to raise the water temperature to 24 and 32 °C. Each water bath housed three 50 x 40 x 10 cm aquariums divided in twenty 10 x 10 x 10 cm individual cells (therefore, each water bath housed sixty 1L cells). Water in the water bath continuously circulated around and underneath all three aquaria by means of a submersible pump. Ten individuals from six streams/populations were randomly placed at each of the two manipulated temperatures (n = 60 individuals per temperature treatment). (TIF) [file pone.0196560.s006.tif]

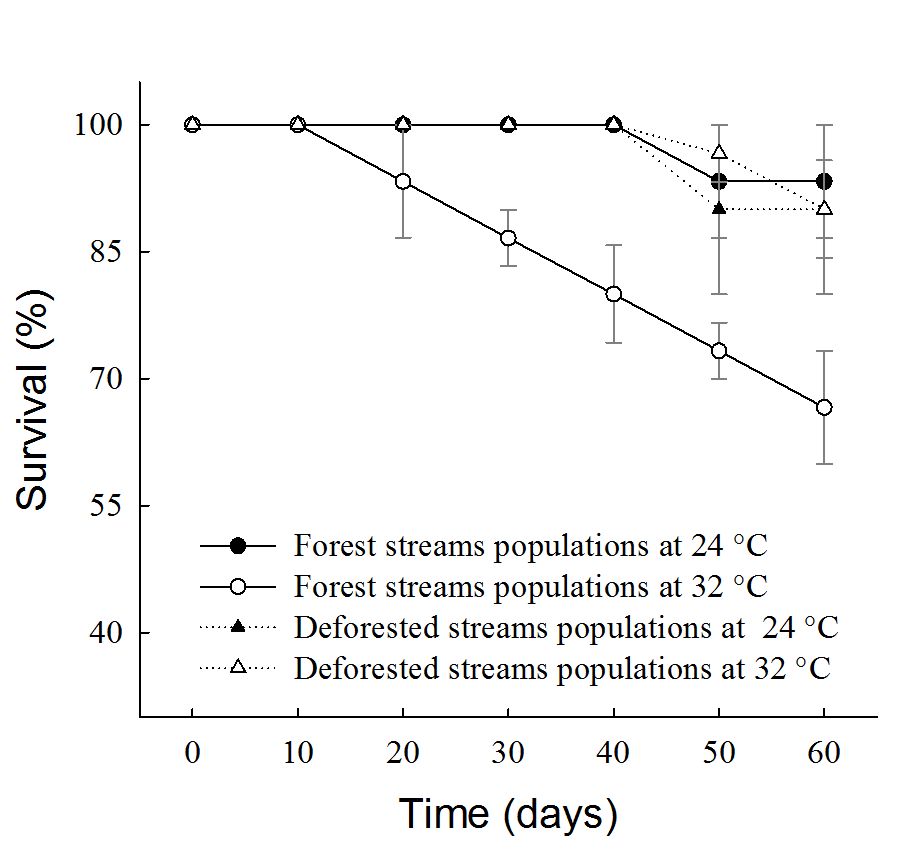

Supplement: S3 Fig — Survivorship of Melanorivulus zygonectes from forest (circles and solid lines) and deforested (triangles and dashed lines) streams populations reared at 24 °C (filled symbols) and 32 °C (blank symbols) during 60 days of experiment. Symbols represent mean values (n = 30 individuals) and error bars represent standard errors. (TIF) [file pone.0196560.s007.tif]
